# Hypothesis: Metformin is a potential reproductive toxicant

**DOI:** 10.3389/fendo.2022.1000872

**Published:** 2022-10-19

**Authors:** Maja Tavlo, Niels E. Skakkebæk, Elisabeth R. Mathiesen, David M. Kristensen, Kurt H. Kjær, Anna-Maria Andersson, Rune Lindahl-Jacobsen

**Affiliations:** ^1^ Faculty of Health Sciences, Department of Epidemiology, Biostatistics, and Biodemography, University of Southern Denmark, Odense C, Denmark; ^2^ Interdisciplinary Center on Population Dynamics, University of Southern Denmark, Odense C, Denmark; ^3^ Department of Growth and Reproduction, Copenhagen University Hospital — Rigshospitalet, Copenhagen, Denmark; ^4^ International Center for Research and Research Training in Endocrine Disruption of Male Reproduction and Child Health (EDMaRC), Rigshospitalet, University of Copenhagen, Copenhagen, Denmark; ^5^ Department of Clinical Medicine, University of Copenhagen, Copenhagen, Denmark; ^6^ Centre for Pregnant Women with Diabetes, Department of Endocrinology, Rigshospitalet, Copenhagen University Hospital, Copenhagen, Denmark; ^7^ Faculty of Health Sciences, University of Copenhagen, Copenhagen, Denmark; ^8^ Department of Neurology, Danish Headache Center, Rigshospitalet - Glostrup, University of Copenhagen, Copenhagen, Denmark; ^9^ University of Rennes, Inserm, École des hautes études en santé publique (EHESP), Irset (Institut de recherche en santé environment et travail) UMR_S, Rennes, France; ^10^ Department of Biology, University of Copenhagen, Copenhagen, Denmark; ^11^ Globe Institute, Section for GeoGenetics, Faculty of Health and Medical Sciences, University of Copenhagen, Copenhagen, Denmark

**Keywords:** metformin, reproductive toxicant, testosterone, endocrine disruptor, environment, wildlife, development

## Abstract

Metformin is the first-line oral treatment for type 2 diabetes mellitus and is prescribed to more than 150 million people worldwide. Metformin’s effect as a glucose-lowering drug is well documented but the precise mechanism of action is unknown. A recent finding of an association between paternal metformin treatment and increased numbers of genital birth defects in sons and a tendency towards a skewed secondary sex ratio with less male offspring prompted us to focus on other evidence of reproductive side effects of this drug. Metformin in humans is documented to reduce the circulating level of testosterone in both men and women. In experimental animal models, metformin exposure *in utero* induced sex-specific reproductive changes in adult rat male offspring with reduced fertility manifested as a 30% decrease in litter size and metformin exposure to fish, induced intersex documented in testicular tissue. Metformin is excreted unchanged into urine and feces and is present in wastewater and even in the effluent of wastewater treatment plants from where it spreads to rivers, lakes, and drinking water. It is documented to be present in numerous freshwater samples throughout the world – and even in drinking water. We here present the hypothesis that metformin needs to be considered a potential reproductive toxicant for humans, and probably also for wildlife. There is an urgent need for studies exploring the association between metformin exposure and reproductive outcomes in humans, experimental animals, and aquatic wildlife.

## Introduction

The oral blood-glucose-lowering drug, metformin, is effective, low-cost and the most commonly used antidiabetic drug in the world (1). Metformin has been used to treat diabetes in European countries since 1958 and is currently recommended as a first-line oral treatment for type 2 diabetes mellitus (T2DM) for both men and women (1, 2). In 2012, metformin was prescribed to more than 150 million people worldwide (3) and was the 4^th^ most prescribed drug in the US in 2019 (4). Unlike insulin, metformin crosses the placenta readily (5) and has the potential to cause negative effects on the developing fetus (6–8). However, metformin is used in pregnant women with T2DM, and benefits on the maternal glycaemic level and neonatal adiposity are demonstrated (9). In addition, there is an increase in experimental studies investigating whether metformin can be used in different diseases and conditions including endometriosis (10). The therapeutic indications for metformin prescription may therefore be expanding, resulting in even more widespread use. However, as stated in a recent review by Triggle et al. (10) metformin may act as an endocrine disrupter through multiple sites of actions and signaling pathways, and this uncertainty may offset the expansion use of metformin.

Recently, we found that offspring of diabetic fathers who were prescribed metformin during the three months before fertilization had an increased risk of malformations, especially in the male sexual organs where malformations were three times more common (11). These findings prompted us to search the literature for indications of other negative reproductive effects of metformin and to present the hypothesis that metformin should be considered a potential reproductive toxicant for humans, and possibly also for wildlife.

### Clinical action, metabolism, and excretion to the environment

**Figure 1:**
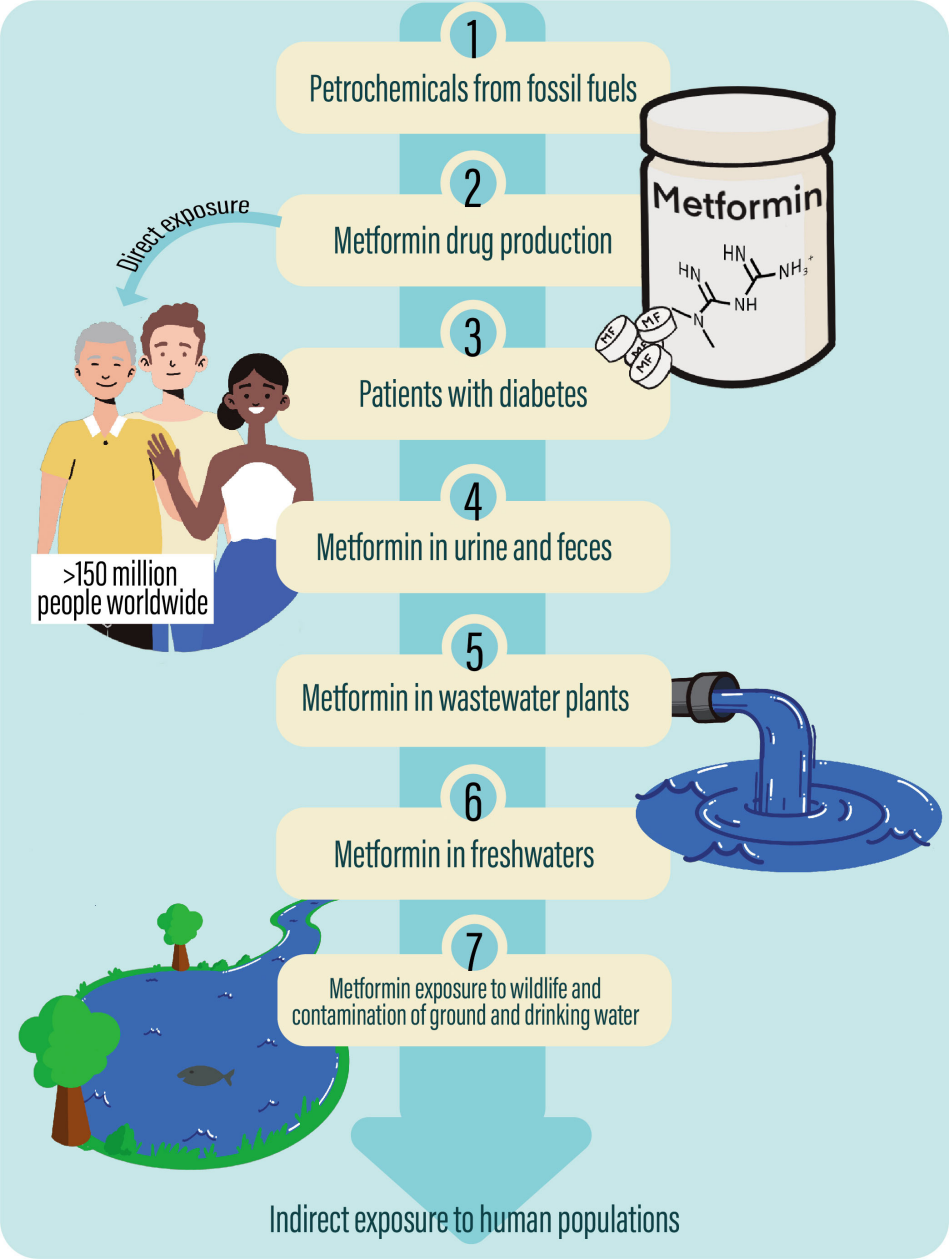
Metformin’s path from the synthesis of the drug from petrochemicals to contamination of the environment *via* wastewater. 1) Metformin derives from petrochemicals, which again derive from fossil fuels such as oil. 2) From petrochemicals there is a production of the drug, metformin which is 3) prescribed to more than 150 million people, including a large proportion of patients with diabetes 2, who thus are directly exposed through therapeutic treatment. 4) As metformin is not metabolized in the human body, it enters the environment unchanged through urine and feces, and 5) metformin thus reaches wastewater treatment plants and later 6) freshwaters as rivers and lakes and drinking water. 7) Metformin, therefore, contaminates our environment affecting the aquatic wildlife and potentially exists as indirect exposure to human populations all over the world.

Evidence suggests that long-term metformin treatment works primarily by inhibiting hepatic gluconeogenesis and secondarily by improving glucose uptake in skeletal muscles and adipocytes, resulting in lowering blood glucose concentrations (12). Metformin presumably affects all tissues within the human body *via* AMP-activated protein kinase (AMPK)-dependent and -independent mechanisms, primarily by inhibition of mitochondrial respiration (13). However, despite more than 60 years of extensive use, metformin’s precise mechanism of action is not known (14). Metformin, like many pharmaceuticals, derives from petrochemicals, which again derive from fossil fuels (15). Unlike most pharmaceuticals, metformin is difficult to decompose and not metabolized in the human body and thus enters the environment unchanged, mainly through urine and feces (16). As metformin enters the aquatic compartments, it can be transformed into guanylurea, and several recent reports provide evidence that both metformin and guanylurea are present in the environment (17) (**Figure 1**). A recent study from 2022, investigating pharmaceutical pollution of the world’s rivers, reported metformin as one of the most frequently detected active pharmaceutical ingredients, as it was detected in over half of the monitored sampling sites (18).

## Reproductive side-effects of male metformin treatment

Our recent nationwide study by Wensink and colleagues (11) showed an association between preconception paternal metformin treatment and genital birth defects in boys in Denmark. In both, the offspring of the background population and of insulin-treated fathers, the prevalence of major birth defects was 3.3%, while the prevalence in the offspring of the metformin-treated father was 5.6% (adjusted OR 1.40, CI95%, 1.08 to 1.82). In the male sexual organs, the malformations were three times more common. The data also supported an association between metformin exposure and alteration in the secondary sex ratios, as the child exposed to metformin, were less often male (49.4%), compared to those without exposure to diabetes drugs (51.4%) or insulin-exposed offspring (51.3%). These findings align with evidence suggesting men exposed to reproductive toxicants may have altered secondary sex ratios (19, 20). However, it is known that hyperglycemia is a risk factor for fetal malformations (21). In the given study a large proportion (84%) of the insulin group likely had been on insulin for many years, whereas we do not know how many in the metformin group were diagnosed and treated during or shortly before spermatogenesis. As it typically takes weeks or months of metformin treatment to control glycemia, the severity of hyperglycemia during spermatogenesis may have differed between the insulin and metformin groups (22). However, if poor glycemic control did play a major role in relation to the increased prevalence of malformation, we would have expected to see a similar signal with an increased prevalence of malformation in fathers taking insulin (23). There is a need for further studies to support our hypothesis, in particular studies accounting for variables such as obesity and glycemic control.

It is well known that men with T2DM have lower testosterone levels. In a recent randomized controlled study, Hu and colleagues (24) found that in men with T2DM, metformin may cause decreasing testosterone levels independent of blood glucose control. The authors reported that a 1-month treatment resulted in a 27.4% (CI95% -47.2 to -7.6) reduction in testosterone levels among men treated with both insulin and metformin, compared with men only treated with insulin. In addition, the authors did another study with a prolonged duration of 3 months of metformin treatment and their results were consistent with the previous study, as they found that compared with the control group, testosterone levels in the metformin group significantly decreased. Despite these studies being rather small in sample, the data strongly suggest that metformin can reduce the level of testosterone to a clinically significant extent in men (24) and metformin may be another reason for the high prevalence of low testosterone in males with T2DM (25). Furthermore, metformin has been used as an anti-androgen for women with polycystic ovary syndrome (PCOS) (26). Together this shows that the compound is a strong disruptor of steroidogeneses in both sexes. Interestingly, in obese males with metabolic syndrome and reduced fertility, metformin has been shown to improve fertility through increased testosterone production (27). In a study, Morgante and colleagues showed that 6-month metformin treatment resulted in an increase in the serum testosterone in obese males (28). However, this may be related to changes in sex hormone-binding globulin (SHBG) in men with metabolic syndrome, treated with metformin, as obesity and metabolic syndrome are associated with lower SHBG concentrations and thus decreased testosterone production, but normal levels of free testosterone (29, 30). However, when obese males are treated with metformin, their metabolic status improves, resulting in increasing SHBG levels, which can trigger testosterone production, as experimental evidence suggests that an increase in SHBG is associated with an increase in testosterone level (31).

Taken together, these data suggest a scenario where the effects of metformin on reproduction need to be seen in the light of the indication for treatment and the effects of the underlying disease.

### Reproductive side-effects of female metformin treatment

In women with PCOS, the effects of metformin treatment are generally described as beneficial, as metformin treatment is associated with a 20% reduction in the testosterone level (26) and improves ovarian cyclicity (27). Furthermore, several randomized trials have shown that metformin treatment in women with PCOS increases clinical pregnancy rates (32).

Metformin is used for the treatment of women with T2DM (9) and currently, there is no evidence that maternal metformin intake is associated with an increased risk of major birth defects. In an international case-control study including 141 malformed offspring, the risk of congenital malformations was regarded as similar in both offspring of women taking metformin for diabetes or PCOS and the background population (33). In addition, a small study on the offspring of women treated with metformin during pregnancy did not find any effects on the testicular size of their sons (34). Thus, in the relatively few and small studies investigating maternal metformin intake during pregnancy, there is found no association with birth defects or other adverse reproductive outcomes. However, studies of large populations of pregnant women using metformin, with a focus on genital malformations, are lacking. There is thus a need for additional studies evaluating the risk of congenital malformations in offspring of mothers treated with metformin. However, it remains clear that metformin in the clinic on one hand is used as an antiandrogenic compound to decrease testosterone in women with PCOS, while it on the other hand is frequently used in women with gestational diabetes or T2DM during pregnancy without considering the potential effects on hormonal regulation of the developing fetus.

### Experimental evidence of reproductive effects of metformin from animal models

**Figure 2:**
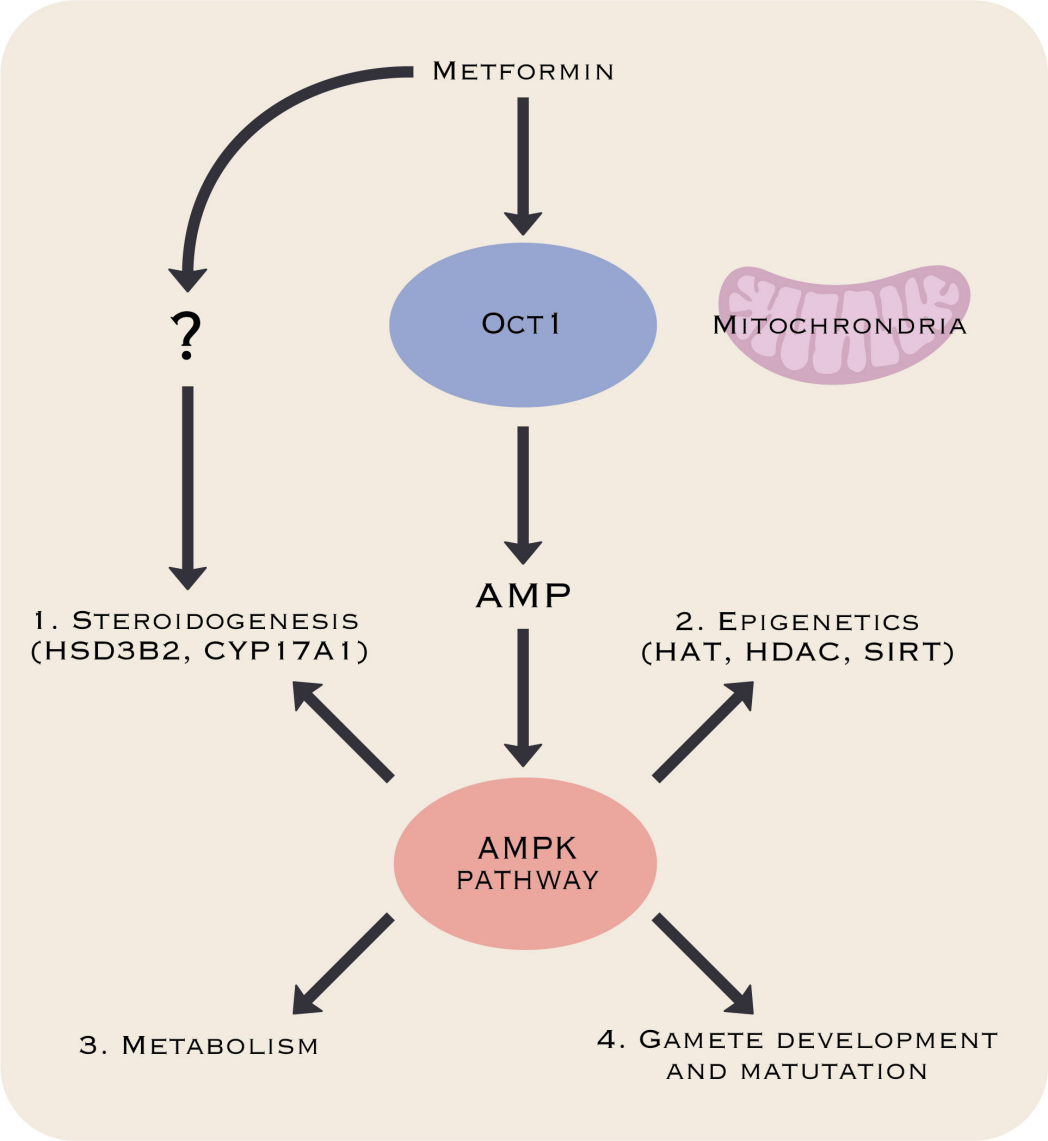
Metformin’s processes of potential interference with fetal development. Metformin may interfere with fetal development through effects on steroidogenesis (38), epigenetics (39), metabolism (40), and gamete development and maturation (27). The mechanism behind these effects remains poorly understood, but data suggest that the AMP-induced protein kinase (AMPK) pathway might play a central role except for effects on steroidogenesis that might be AMPK-independent (38).

Disruption of e.g., hormonal regulation during fetal life can have adverse health outcomes later in life (35). Hence, exposure to drugs during sensitive periods of sexual differentiation can induce alterations in cell numbers, leading to irreversible reductions in sperm and oocyte production that ultimately can influence fecundity later in life (36, 37). Evidence from experimental studies suggests that metformin can interfere with fetal life through at least four essential processes: (*i*) steroidogenesis (38), (*ii*) epigenetics (39), (*iii*) metabolism (40), and (*iv*) gamete development and maturation (27) (**Figure 2**). The exact mechanism of metformin’s actions remains to be completely understood, however, it is clear that metformin inhibits Complex I of the mitochondrial respiratory chain at relatively high concentrations (mM) (27). This inhibition results in a decline in ATP production by mitochondria and activation of the AMPK pathway. It has been suggested that the effects on epigenetics, metabolism, and gamete development might be through AMPK, as the signaling pathway is a crucial cellular energy sensor that maintains cellular energy homeostasis. However, not all effects can be explained by activation of the AMPK pathway and the effects on steroidogenesis have been suggested to be AMPK-independent (38).

The potential effects on fertility have especially received much attention and it is clear that maternal exposure to metformin can interfere with reproductive parameters in male offspring. In a study investigating the effect of *in utero* and lactational exposure to metformin in male rat offspring (41), the authors reported a significant decrease in the number of spermatids and spermatids per organ, as well as daily sperm production in male rat offspring exposed to metformin during gestation and lactation, compared to controls. Interestingly, the decrease in sperm count was only observed in the offspring of mothers exposed to metformin during both gestation and lactation, suggesting that the exposure is needed to cover the entire critical period of male sexual differentiation in the rat.

In another study, Tartarin et al. (42) reported that metformin can reduce testosterone production *in vitro*, and probably also *in vivo*. The authors reported that *in vitro*, metformin decreased the secretion of testosterone by human fetal testicular tissue at a therapeutic dose (50 μM) by 45% and found a reduced testicular size and Sertoli cell population *in vivo*. The authors thus suggest that metformin can alter the masculinization of human offspring when mothers are exposed during pregnancy. Furthermore, the authors reported that the testosterone secretion by mouse fetal testes was reduced by 20% at a concentration of 500μM. Furthermore, the authors reported that metformin decreased mRNA expression of the main factors involved in steroidogenesis by 60-90% in mouse fetal testis. *In vivo* exposure of metformin to mice during pregnancy reduced the size of both fetal and neonatal testes of offspring. The number of Sertoli, but not germ cells, was slightly increased in both the fetal and neonatal period and the Leydig cell population was reduced in the fetal period.

Taken together, this evidence suggests that metformin may have anti-androgenic effects and that it might influence the development of the male reproductive tract and thus alter male fecundity later in life (43).

The direct effect on fertility is supported by a study from 2021, where Faure et al. (44) reported that metformin exposure *in utero* induced sex-specific metabolic and reproductive changes in adult rat offspring. Adult males exhibited reduced fertility, manifested as a 30% decrease in litter size compared to controls whereas adult females presented no clear reduction in fertility. In this study the lower fertility in male rats was not due to changes in sperm production or motility, but rather to lower sperm head quality, including significantly increased spermatozoa head abnormality with greater DNA damage. The authors suggested that metabolic modification by metformin may alter the expression of epigenetic regulators, which could contribute to reduced fertility. It has been suggested that the predominant mechanisms behind the effects leading to decreased fertility are likely through the AMPK pathway, while the effects on androgen production regulating key steroidogenic enzymes HSD3B2 and CYP17A1are likely through an AMPK independent pathway (38).

### Metformin in the environment

The expanded metformin use and its resistance to decompose may have an influence on the environment. Several studies have investigated the presence of metformin and guanylurea in our environment, and the results are unambiguous: Metformin is ubiquitous in the aquatic environment, all over the world. In a systematic review, Ambrosio-Albuquerque, and colleagues (45) reported measurable concentrations of metformin in several different aquatic sources, including influent, sludge, and effluent from wastewater treatment plants, sewage, different surface waters, e.g., rivers, lakes, and oceans, drinking water and sediment. The percentage of detection of metformin varied across the different sources, ranging from e.g., 8% for drinking water, 28% for surface waters, and 51% for urban wastewater. In addition, a study from 2022, investigating active pharmaceutical ingredients in 258 of the world’s rivers, from 1052 locations, in 104 countries, detected metformin at over 50% of the sampling sites. The authors reported a similar frequency of metformin across continents, and metformin was one of the pharmaceuticals analyzed, which was present in the highest concentrations (18). In fact, metformin has been recognized as the most frequently detected anthropogenic-organic contaminant in the aquatic environment among several analyzed pharmaceuticals in different studies and is considered an emerging pollutant of concern (45).

As active pharmaceutical ingredients, such as metformin, are biologically active molecules, specially designed to interact with several biochemical pathways within the human body (18), the widespread presence of metformin in the aquatic environment should be of concern. Notably, metformin is also found in several sources of drinking water around the world, meaning that numerous people worldwide potentially are exposed. Despite the removal of metformin in wastewater treatment plants having a high efficacy rate, ranging from 84% to 99% (46), metformin, and guanylurea are still widely detected in both surface- and drinking water. Another concern is metformin’s chlorination byproducts. Chlorine is used for the disinfection of drinking water all over the world, and there is evidence that chlorine can oxidize metformin into two byproducts: Y; C_4_H_6_C_1_N_5_ and C; C_4_H_6_C_1_N_3_ (47). Evidence suggests that disinfection byproducts formed during chlorine disinfection have a larger negative effect on human health compared to their parent compounds (48). The byproduct C has been detected in 68.40% of tap water in 32 cities in China (48). In addition, in a recent study (49) byproduct C was detected in urban drinking water from multiple countries, including China and the US, and it was demonstrated that the production of both byproduct C and Y could be increased with increasing metformin concentration exhibiting marked toxicities of a potential health concern and thereby being a hidden threat to the global water supply. So, although the current levels of metformin present in drinking water are not regarded as a direct health concern to humans, the potential threats of metformin’s chlorination byproducts should be explored further (49).

### Effects on aquatic wildlife

As metformin is detected widely in the aquatic environment, it is important to consider whether this constitutes a threat to aquatic wildlife. Several studies report that metformin exposure in environmentally relevant concentrations can cause potential endocrine disruption in fish. Niemuth and Klaper (50) reported that exposure to metformin in a concentration relevant to wastewater effluent levels (40 μg L^-1^), caused the development of several alterations in male fathead minnows, including a significantly higher occurrence of intersexuality, compared to control males. Furthermore, they reported a significant reduction in overall size in metformin-treated males as well as significantly fewer cumulative clutches laid per mating pair over time and mean clutch size for metformin-treated males, compared with controls. In contrast, in a fish model using Oryzias latipes, Lee et al. (51) reported no intersex in male gonads, but the occurrence of intersex in F0 generation female gonads in a dose-dependent manner was found.

Additionally, they found that among F0 generation male fish, metformin significantly increased gene expression of both CYP19a and estrogen receptor α. Among F0 generation female fish metformin significantly decreased the expression of ERβ1 and VTG2. Among the F1 generation, metformin significantly increased the expression level of estrogen receptor α in female fish, and significantly decreased the expression of VTG1 in male fish. These sex-specific effects indicate that metformin exposure may cause feminization in male fish and deactivate the reproductive system in female fish (51). Several other studies have investigated the association between metformin and alterations in the expression of specific genes related to reproduction in fish models (52, 53). Niemuth and Klaper (52) have provided evidence that metformin may be an endocrine disrupter, as they showed that among fathead minnows exposed to 40 μg L^-1^ metformin for a year, there was an upregulation of the expression of five endocrine-related genes (AR, 3β-HSD, 17β-HSD, CYP19A1, and SULT2A1) in male gonads tissue. Furthermore, they reported a significant correlation between the expression of three endocrine-related genes (3β-HSD, 17β-HSD aCYP19A1) in the testis and the occurrence of intersex in the gonads. In addition, a significant upregulation of mRNA encoding for VTG in metformin-treated male fish, compared with controls has been reported (53).

Taken together, the experimental findings suggest that metformin can interfere with not only fecundity in mammals but also can act as a disruptor of sexual development in fish at environmentally relevant concentrations. However, the bioaccumulation of metformin in surface water worldwide results in human exposure and is of emerging concern. However, the effects of chronic exposure are poorly understood and need future attention for evaluating the consequences of the increasing amount of metformin found in the environment (45, 54).

## Discussion and conclusion

Evidence is accumulating that metformin, besides its well-documented glucose-lowering effects, may act as a reproductive toxicant in humans, experimental rodents, and fish. We recommend that the adverse reproductive effects of metformin should be examined further. Particularly, there is an urgent need for studies exploring the association between metformin exposure and reproductive outcomes in humans and experimental animals concerning the safety of the offspring following parental metformin treatment. The study by Wensink and colleagues (11) on paternal metformin intake should be repeated in another cohort. Clinical studies in normal and diabetic men investigating the impact of metformin on sex hormones are urgently needed. Furthermore, the effect of maternal exposure to metformin in early pregnancy on the development of congenital malformations and its impact on offspring should be investigated. In experimental animals, a randomized study on intrauterine metformin exposure and e.g., the anogenital distance in male offspring can help shed light on metformin’s antiandrogenic effects and reproductive toxicity. In addition, to gain insight into the underlying mechanisms of metformin on steroidogenesis and whether these in fact are AMPK-independent, experimental studies using AMPK-knockout mouse models can be executed. If our hypothesis that metformin is a reproductive toxicant is supported, alternative drugs for the management of T2DM must be considered. Furthermore, evidence of a widespread presence of metformin in the aquatic environment raises concern. Ubiquitous exposure to metformin may not only be considered a potential threat to aquatic wildlife, but also to humans and wildlife in general through continuous exposure from drinking water. As metformin has been used to treat diabetes since 1958 and is difficult to decompose, we speculate that metformin might be accumulated in sedimentary deposits over the latest 60 years, especially in coastal environments associated with river outlets. This is a potential huge reservoir for metformin pollution close to densely populated urban areas in the world.

## Data availability statement

The original contributions presented in the study are included in the article/supplementary material. Further inquiries can be directed to the corresponding author.

## Author contributions

MT, RL-J, and NS have made substantial contributions to the conception and design of the work. MT, RL-J, NS, DK, and EM have made substantial contributions to the acquisition, analysis, and interpretation of data/literature. MT, RL-J, NS, EM, DK, A-MA, and KK have been drafting the work or revising it critically for important intellectual content. All authors have approved the final version of the paper to be published and have agreed to be accountable for all aspects of the work in ensuring that questions related to the accuracy or integrity of any part of the work are appropriately investigated and resolved. All authors contributed to the article and approved the submitted version.

## Funding

The authors acknowledge an unrestricted grant from Ferring. The funder was not involved in the study design, collection, analysis, interpretation of data, the writing of this article or the decision to submit it for publication.

## Acknowledgments

We are grateful for unrestricted grant support from Ferring.

## Conflict of interest

The authors declare that the research was conducted in the absence of any commercial or financial relationships that could be construed as a potential conflict of interest.

## Publisher’s note

All claims expressed in this article are solely those of the authors and do not necessarily represent those of their affiliated organizations, or those of the publisher, the editors and the reviewers. Any product that may be evaluated in this article, or claim that may be made by its manufacturer, is not guaranteed or endorsed by the publisher.
